# Caries Etiology and Preventive Measures

**DOI:** 10.1055/s-0043-1777051

**Published:** 2024-03-31

**Authors:** Frederic Meyer, Erik Schulze zur Wiesche, Bennett T. Amaechi, Hardy Limeback, Joachim Enax

**Affiliations:** 1 Research Department, Dr. Kurt Wolff GmbH & Co. KG, Bielefeld, Germany; 2 Department of Comprehensive Dentistry, School of Dentistry, University of Texas Health San Antonio, San Antonio, Texas, United States; 3 Faculty of Dentistry, University of Toronto, Toronto, ON, Canada

**Keywords:** caries, teeth, plaque, toothpaste, fluoride, hydroxyapatite, prevention, oral care

## Abstract

Caries is a widespread disease in both children and adults. Caries is caused by the conversion of fermentable carbohydrates by plaque bacteria into acids on the tooth surface. Thus, it is important to focus on sugar reduction and plaque control. For efficient plaque removal/control, state-of-the-art toothpastes contain various active ingredients such as antimicrobial agents (e.g., chlorhexidine, stannous salts, and zinc salts), abrasives (e.g., calcium carbonate, calcium phosphates, and hydrated silica), surfactants (e.g., sodium lauryl sulfate and sodium methyl cocoyl taurate), and natural compounds (e.g., polyphenols and xylitol). Agents with pH-buffering and calcium-releasing properties (e.g., calcium carbonate and calcium phosphates) and biomimetic actives (e.g., hydroxyapatite) reverse the effects of the acids. Additionally, modern toothbrushes (i.e., electric toothbrushes) as well as dental floss and interdental brushes significantly help remove plaque from dental surfaces including interproximal surfaces. In conclusion, modern concepts in caries prevention should focus not only on tooth remineralization alone but also on the control of all the key factors involved in caries development.

## Introduction

Teeth are highly mineralized hydroxyapatite-based tissues having a hierarchically organized nano- and microstructure. 1 2 3 Human teeth are, additionally, organized in hydroxyapatite prisms that lead to an increased resistance against wear. 3 Because of that, teeth are hard and resist many different mechanical, thermal, and chemical influences. Since the mineral phase of human teeth consists of mainly hydroxyapatite, a calcium phosphate mineral, it can be partially or fully dissolved in acids. 4 The dissolution process of hydroxyapatite, induced by acids of bacteria origin in the plaque, leads to dental caries. 5 The pH in plaque can drop to about 4.3 to 4.8, which slowly dissolves tooth structure. 6 It is known that even densely packed enamel has a critical pH of 5.5. However, the pH is not the only crucial factor for tooth crystal dissolution because this process depends also on the concentration gradients of calcium and phosphate ions in saliva and plaque. 7 Dental plaque is a mixture of living and dead microorganisms, extracellular polysaccharide matrix (EPS) produced by these microorganisms, saliva-derived components, and food debris. The presence of dental plaque, when it shifts to a cariogenic composition, is the main cause for dental caries when there is a source of fermentable carbohydrate.

Caries can be defined as “the results—the signs and symptoms—of a localized chemical dissolution of the tooth surface caused by metabolic events taking place in the biofilm (dental plaque) covering the affected area.” 5 This is in contrasts to the process of “dental erosion” where the tooth demineralization occurs with acids not derived from plaque bacteria but from acidic food, beverages, etc. 8 9 Clinically, dental caries and dental erosion have different appearances and outcomes on the teeth.

Under healthy conditions, calcium and phosphate ions from saliva remineralize the tooth surfaces constantly, which is in an equilibrium with constant demineralization of the tooth surfaces caused by bacterial acids or acids derived from diet. However, although supersaturated with Ca ^2+^ and PO _4_^3−^ ions in a bioavailable form, saliva remineralization process is slow and insufficient to tackle the caries process alone. 10 Moreover, in mature thick and sticky plaque, as seen in poor oral hygiene conditions, it takes a long time for saliva and salivary components to penetrate through the dense layer to reach the tooth surface to induce remineralization. Sucrose and other monosaccharides are readily taken up by plaque bacteria and metabolized to organic acids. These acids will slowly dissolve enamel crystallites. In high-risk patients, such as patients undergoing an orthodontic therapy, clinically visible caries lesions can develop and be diagnosed within just 1 month. 11

Batchelor and Sheiham analyzed the caries susceptibility of different tooth surfaces in children aged 5 to 16 years in the United States. They found that “the most susceptible group consists of six tooth surfaces: the buccal pits and occlusal fissured surfaces of the first molar teeth. The second group consisted of 12 sites on the second molar and premolar teeth. The group formed by the least susceptible sites included the largest number of tooth surfaces and consists of the majority of the lower anterior teeth and canines.” 12 Additionally, the so-called early childhood caries (ECC) mainly affects the upper incisors. 13 14 However, it should be emphasized that all teeth can be affected by caries. What all tooth surfaces that are often affected by caries have in common is that the dental plaque removal seems to be insufficient.

Caries is a dental disease affecting billions of individuals around the globe and having a huge negative impact on the global economy. 5 14 15 16 The economic burden of dental caries is estimated to be approximately US$298 billion per year on treatment costs and US$144 billion per year as productivity loss. 17 For example, data on the mean caries experience of the German population are presented in Table 1 . Overall, the caries experience based on the number of teeth affected in the German population, where high oral health standards are established, is lower compared to other countries; however, almost all adults had experienced caries. 18

**Table 1:** Mean extent of caries experience and mean caries prevalence of different age groups in Germany (measured using the decayed, missing, and filled teeth [DMFT] index)

Age group (y)	Mean extent of caries experience	Mean caries prevalence (%)
12	0.5 teeth	18.7
35–44	11.2 teeth	97.5
65–74	17.7 teeth	99.9
75–100	21.6 teeth	99.7

Source: Data taken from Jordan and Micheelis. 18

Compared to other countries, the prevalence of dental caries is similar. For example, data from the National Institute of Dental and Craniofacial Research show that there is only a minor decline in the dental caries experience among all age groups in the United States ( Table 2 ).

**Table 2:** Mean prevalence of caries experience (measured using the decayed, missing, and filled teeth [DMFT] index) of different age groups in the United States

Age group (y)	Mean prevalence of caries experience, 1999–2004 (%)	Mean prevalence of caries experience, 2011–2016 (%)
20–34	85.7	82.0
35–49	94.2	92.5
50–64	95.6	96.4

Source: Data taken from National Institute of Dental and Craniofacial Research. 19

Every person is prone to dental caries at some time in their life. There are also several patient groups that are especially at high risk of developing caries, such as the following:

- *Children and adolescents:* Diet might contain higher sugar amounts, tooth brushing time is limited, and plaque removal is inefficient. Not only the amount of sugar but also the frequency is important. Tooth brushing technique might not be sufficient for efficient plaque removal. 14
- *Patients undergoing orthodontic therapy:* Orthodontic appliances, especially braces and brackets, make it difficult for patients to remove plaque appropriately. Brackets increase dental plaque buildup, which increases caries risk. 11
- *Patients suffering from hypomineralized teeth:* There are several known conditions that may cause developmental hypomineralization of teeth. Molar hypomineralization, until recently often called molar incisor hypomineralization, is one of the most prevalent developmental defects of tooth enamel (DDE). However, all affected persons have one thing in common, which is tooth sensitivity. This leads to diminished brushing of the teeth and reduced plaque removal. Additionally, hypomineralized tooth structures are prone to develop caries, as the mineral content is deficient and enamel integrity is not intact. Following this, acids can dissolve less mineralized enamel faster than healthy enamel. 20
- *Patients with reduced salivary flow (hyposalivation):* Saliva protects and remineralizes teeth. While saliva helps reduce the cariogenicity of sugars, a reduced salivary flow results in diminished antibacterial effects because of the inefficient dilution of acids, diminished buffering capacity, and lowered remineralizing potential. Dental plaque buildup is increased. Taken together, this leads to an increased caries risk of the affected persons. 5
- *Elderly with exposed dentin:* Exposed dentin is more prone to developing dental caries as the mineral content and density of dentin is reduced compared to enamel. Also, the critical pH for dentin dissolution is 6.2 (unlike that of enamel, which is pH 5.5), so dentin demineralizes easily when exposed. Additionally, elderly people suffer in many cases from hyposalivation and might not be able to brush their teeth properly, due to physical limitations. This type of dental caries is also known as root caries. 21

Taken together, measures to prevent caries or to reduce the risk for caries development are important for all age groups and all patient groups. Even though one of the above-mentioned might only occur in a certain (short) period of life, dental caries development can only be halted and reversed in the early phases. 22

The present review is focused on the primary etiological factors in caries development, with highlight on the modern clinical preventive strategies that can counteract the same. Special emphasis will be given to modern (as well as biomimetic) approaches and state-of-the-art concepts as well as the active ingredients to control and reduce plaque considering that the correlation of the presence of plaque and caries is well known. Although this review is focused on the main caries risk factors, it is important to acknowledge the influence of secondary risk factors such as environmental factors and personal factors, which include, but are not limited to, social determinants for oral health (education, sociodemographic status, and income), dental knowledge, access to dental care, attitudes, behaviors, and personal/cultural beliefs. While there are review articles on caries etiology and preventive measures available in the literature, no review article has focused on various approaches as well as on different toothpaste compounds (including biomimetic hydroxyapatite) for caries prevention. Therefore, the aim of this review article is to give a state-of-the-art overview of various modern active ingredients and strategies to prevent dental caries, with a special focus on removal and control of dental plaque.

## Methods

This publication was written as a narrative review. All the authors individually searched for literature based on common knowledge. Publications and textbooks were used for manuscript preparation. PubMed database was used as the primary source for scientific publications. All the authors reviewed the literature that was chosen for this publication and suitable original articles were included.

## Caries Etiology

### General Considerations

**Fig. 1:**
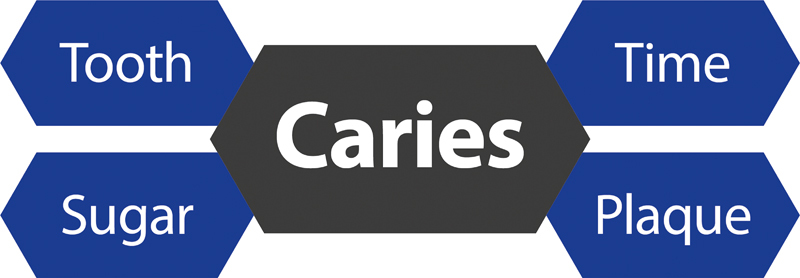
Schematic overview on the causes of caries. While many caries preventive concepts focus on the tooth (i.e., remineralization and inhibition of demineralization), addressing the two main factors involved in caries development (sugar reduction and plaque removal) is equally important to reduce the individual's caries risk (for details see the text).

Caries can affect enamel, dentin (including roots of teeth), and cementum. 5 Due to the lower degree of mineralization as well as a lower proportion of hydroxyapatite and a less complex nano- and microstructure, dentin and cementum are more susceptible to a caries attack than the enamel. Dental caries can only develop when bacteria in the plaque on tooth surfaces take up and ferment carbohydrates (“sugars”), leading to acid production. Dental caries is known and defined as a process that occurs over time ( Fig. 1 ). 23

Out of different carbohydrates, sucrose and glucose have the highest cariogenic potential. Less cariogenic are maltose, lactose, fructose, and starch. 24 The sugar alcohol, xylitol, on the other hand, has caries preventive properties. 25 It needs to be considered that both the total intake and frequency of sugar intake play an important role for caries development. Studies have shown that the total amount of sugar intake might be more relevant than the frequency of sugar intake. 26 Several model systems have studied sugar uptake of different bacteria. Sugars, such as glucose, can be utilized efficiently by the bacteria through two pathways. Sugar can be used by cariogenic bacteria to produce extracellular glycan, a sticky polysaccharide that serves as a reservoir for sugar molecules; it is used for adherence to tooth structure and contributes to adherence of other oral bacteria to contribute to biofilm formation. This is the extracellular pathway of sugar utilization. On the other hand, the bacteria can take the glucose into its cytoplasm for metabolism for the intracellular pathway. Cariogenic bacteria then use mostly phosphotransferase and proton motive force to internalize carbohydrates. Inside the cell, the sugars will be used by the bacteria for energy production through the glycolic pathway. The metabolic product derived from the glycolysis, apart from energy production in the form of adenosine triphosphate, is pyruvate. Pyruvate is used in the bacteria cell for NAD ^+^ (nicotinamide adenine dinucleotide) production. The reduction of pyruvate leads to the production of lactic acid, which is extreted. 27

### Dental Plaque

It is known that “dental plaque is the community of microorganisms found on a tooth surface as a biofilm, embedded in a matrix of polymers of host and bacterial origin.” 28

Studies on dental plaque revealed more than 700 different bacterial species belonging to the oral microbiome. 29 Further research has also investigated the oral mycobiome and virome. 30 Nowadays, it is well known that changing the biochemistry of the oral environment, such as changing diet, leads to a shift in the microbiome. Besides the presence of bacteria in the oral cavity in saliva and on the oral mucosa, they are enmeshed in and organized in the plaque. Plaque is a mixture of several bacteria, other microorganisms, and also viruses that are interacting with each other, while they have EPS as protective surroundings. 31 32 In addition, plaque also contains derivates from saliva and diet. Plaque, when not removed, can be mineralized and will turn into dental calculus. In a condition of constant and frequent sugar intake, some of these bacteria are highly adapted to take up those sugars for their energy production and cell metabolism. 33 Those are, among others, *Streptococcus* spp., but also *Prevotella* spp. and *Veillonella* spp. 28 34 35

Bacteria that take up and metabolize sugars to acids in plaque are known as acidogenic (acid-producing) bacteria. Those bacteria that can survive acidic conditions are known as aciduric bacteria. Both types of bacteria play an important role in the process of caries development. 36 While acidogenic bacteria (that are usually aciduric as well) produce an acidic environment, aciduric bacteria help maintain the plaque biomass within which the acids will stay on the tooth surface. Recurrent acid attacks and inconsistent plaque removal lead to dental caries.

Prominent bacteria and fungi that are present in plaque and involved in the caries process are the following: 24 37 38

- *Streptococcus mutans* and other low-pH streptococci.
- *Rothia* spp.
- *Actinomyces* spp.
- *Lactobacillus* spp.
- *Bifidobacterium* spp.
- *Candida albicans* .
- *Selenomonas sputigena* .

**Fig. 2:**
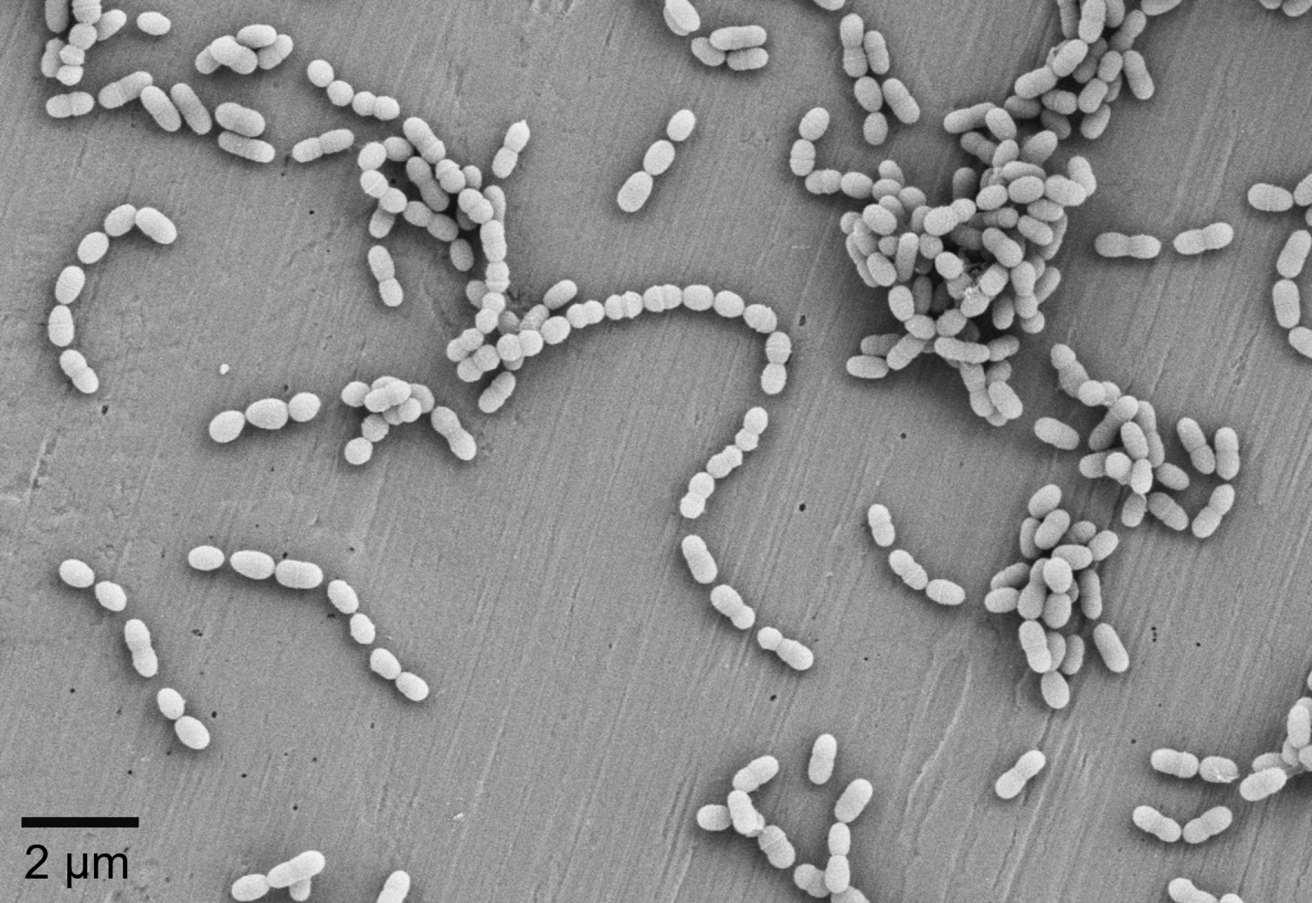
Scanning electron microscopy image of *Streptococcus mutans* .

One of the most prominent microorganisms that is involved in the process of dental caries is *S. mutans* . However, studies have found out that the presence of *S. mutans* is not necessarily connected to dental caries. It could also be shown that dental caries can develop in the absence of *S. mutans* . 39 A microscopic image of *S. mutans* can be found in Fig. 2 .

### Caries Development on the Microscopic Level

**Fig. 3:**
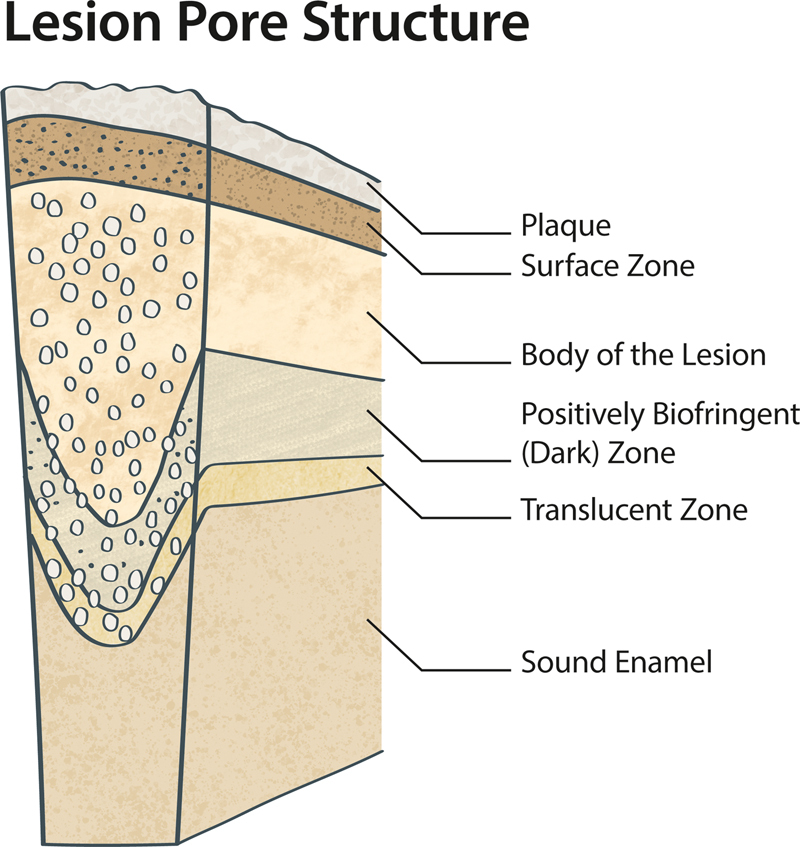
Schematic view on a caries lesion in the enamel with different “zones.” (Adapted from Robinson. 40 ).

The mechanisms of caries development on the microscopic level are highly dynamic and remain complex and, to date, have not been completely understood. Robinson et al reviewed the literature in this field. 40 It was concluded that there are four different “zones” in enamel caries lesions ( Fig. 3 ) 40 :

- Surface zone.
- Body of the lesion.
- Positively birefringent (dark) zone.
- Translucent zone.

These zones differ in pore structure and mineral composition. The lowest mineral density in an enamel caries lesion can be found in the “body of the lesion” zone. This zone also has the highest porosity (∼25–50%). 40 The body of the lesion zone is of high importance for the caries process. While the surface zone of the lesion will recover/remineralize through calcium and phosphate ions derived from the saliva, the lesion body will remain. The demineralization process can continue here in the presence of acids.

The caries process involves the dissolution of the tooth hydroxyapatite with the consequent release of calcium and phosphate ions. In a study using transmission electron microscopy, it was shown that defects in the hydroxyapatite lattice can be considered the starting point for the demineralization (dissolution) of hydroxyapatite. 41

From a chemist's viewpoint, the demineralization of hydroxyapatite can be simplified as follows: 42

Dental apatite + acid → Ca ^2+^ + HPO _4_^2−^ + PO _4_^3−^ .

Based on these released ions, many different reactions can occur in the oral cavity, mainly determined by the local pH as well as by the available ions (e.g., Mg ^2+^ , CO _3_^2−^ , OH ^−^ ) and their concentrations. For example, it has been reported that dicalcium phosphate dihydrate, CaHPO _4_ · 2 H _2_ O, as well as magnesium-substituted tricalcium phosphate, (Ca,Mg) _3_ (PO _4_ ) _2_ , can be formed. 42 In addition, initially formed phases can further be transformed to other phases. 42 Thus, on the chemical and crystallographic level, the caries process is highly complex.

### Caries Detection

Caries detection for public health measures or clinical trials is often performed by using the decayed, missing, and filled teeth/surface (DMFT/S) index or by using the International Caries Detection and Assessment System (ICDAS). ICDAS offers several advantages such as the detection of early caries lesions (initial caries). 43 Furthermore, monitoring caries lesions (surveillance) is possible when using ICDAS. It is pertinent to mention that data collected with ICDAS can be converted into the DMFT/S index. The codes have been used in several clinical trials and can be seen as standard measure for the same 44 45 :

- Code 1: First visual change in enamel.
- Code 2: Distinct visual change in enamel.
- Code 3: Localized enamel breakdown because of caries with no visible dentin or underlying shadow.
- Code 4: Underlying dark shadow from dentin with or without localized enamel breakdown.
- Code 5: Distinct cavity with visible dentin.
- Code 6: Extensive distinct cavity with visible dentin. 40

Visual caries detection using DMFT/S or ICDAS can be combined with bitewing radiographs or other techniques such as near-infrared light transillumination of dental tissues. 44 46 In *in vitro* and *in situ* studies, caries lesions in enamel and dentin can be analyzed with different analytical techniques with high resolutions that cannot be performed under *in vivo* conditions. Examples are microradiography and different hardness tests. 47

## Preventive Measures

### General Considerations

As described earlier, one of the three crucial factors for dental caries development needs to be controlled for prevention of caries to be successful. The three factors are sugar (change of dietary habit), plaque (improvement of plaque removal), or time (either frequency of sugar intake or time between toothbrushing and plaque removal). Following this, prevention programs for oral health should be based on scientific data and also focus on diet and nutrition consultation. 48

A general overview of the mechanisms for reducing the overall caries risk is presented in Table 3 .

**Table 3:** Overview of different strategies to prevent caries

Target	Intervention
Plaque	• Oral hygiene • Tooth brushing • Antimicrobial agents • Abrasives
Diet/sugars	• Reduction of sugar intake • Stimulation of salivary flow • Neutralization of plaque acids with water
Dental tissues	• Remineralization agents • Toothpastes, gels, and mouthwashes (home use) • Varnishes (professional use)

Source: Modified from Limeback. 24

Although many strategies in preventive oral health care are based on remineralization concepts, it is also important to remove/control plaque because this in combination with fermentable carbohydrates is the main factor for the development of caries. Controlling oral bacteria in plaque means they would not grow too fast or are constantly reduced in the total cell count and that the composition stays in a healthy state (bacterial homeostasis).

### Dietary Control of Caries

It is important to discuss with the children and parents on the frequency and total amount of sugar intake and a reduction of sweet beverages. Sweet beverages, especially the acidic ones, coupled with reduced oral hygiene, are one of the main causes of ECC and dental caries in the primary dentition. 14 However, the permanent dentition is also prone to caries when sweet acidic beverages are consumed. Globally, there are many attempts to reduce the sugar intake such as taxing sugars, dietary advises in school, etc. (for more information on dietary control and caries prevention, see, e.g., Al-Dajani and Limeback 49 ). Nevertheless, complete elimination of sugary drinks is often not the key for the individual understanding of the correlation between sugar intake and dental caries. Continuous guidance is more important for a long-lasting success in the individual caries prevention. Therefore, sweet beverages and candy can be advised to be consumed with main meals, followed by drinking water and tooth brushing. Benefits of sugar reduction and healthy diet on overall health can also be mentioned for a better general understanding.

### The Role of Fluoride

When it comes to caries prevention, fluorides have been used as an anticaries agent in oral care for decades. 50 51 Interestingly, however, incorporation of fluoride into the tooth surface is very low (just a few micrograms per square millimeter, only at the outermost tooth surface) and no “hardening” of the tooth can be expected. 52 Thus, other modes of action of fluorides seem to be predominantly for the caries protective effect of fluorides. Those remineralizing properties of fluorides have not yet been fully understood. The same can be stated for its mechanisms of inhibiting demineralization. Nevertheless, there is a limit to the application of fluorides as described by Hausen et al. 53 The addition of fluorides from different sources does not enhance the anticaries effect. In contrast, the risk of chronic fluoride intoxication will be increased with the use of different fluoride sources. 54 The most prominent visible sign for fluoride intoxication is dental fluorosis. The prevalence of dental fluorosis ranges approximately between 20 and 65%, depending on the country and the use of fluorides in water, salt, milk, and oral care products. 55 56 It is important to note that fluoride has antimicrobial effects on oral bacteria. 57 58 For example, fluoride inhibits various bacterial enzymes that are necessary for cell growth, sugar transport, and energy metabolisms (e.g., enolase, F-ATPase, sulfatase, catalase, phosphatases, and phosphoglucomutase).

### Mechanical Removal of the Plaque

Plaque removal is achieved by using toothbrush in combination with toothpaste. While studies have shown that plaque growth in the oral cavity for 72 hours can be removed without the use of a toothpaste, 59 these results cannot be transferred to real-life situations. Toothpastes contain abrasives that are needed to remove not only dental plaque but also stains from the tooth surfaces. With respect to the toothbrush choice, both electric and manual toothbrush can remove dental plaque efficiently. However, the toothpaste user is crucial. With regard to oral hygiene in cases of low-motivated individuals, it has been shown that electric toothbrushes are more efficient than manual toothbrushes. 60 Different bristle types and stiffness of bristles can be used to improve the cleaning efficiency of the respective toothbrushes. 61 62 The mechanical plaque removal by toothpastes is based on abrasives and is supported by surfactants and antimicrobial agents. Commonly used abrasives in modern toothpastes are hydrated silica, calcium carbonate, or calcium phosphates ( Table 4 ). 50 63 In contrast to the toothbrush filaments (millimeter scale), state-of-the-art toothpaste abrasives such as hydrated silica have a specific microscopic structure (nanometer to micrometer scale) that can help clean even pits and fissures. 64 Calcium-containing toothpaste abrasives (e.g., dicalcium phosphate dihydrate, calcium carbonate, calcium pyrophosphate, and hydroxyapatite) can also support the tooth remineralization process. Abrasives that are soft or have a medium hardness are often preferred since they are not harmful to exposed dentin and gingiva. In general, the abrasion (and consequently the cleaning efficacy) is increased with increasing concentration of abrasives. 64

**Table 4:** Overview of abrasives in modern toothpastes for the mechanical removal of plaque 63

Abrasives	Chemical formula	Relative hardness
Sodium bicarbonate	NaHCO _3_	Soft
Dicalcium phosphate dihydrate	CaHPO _4_ · 2 H _2_ O	Soft
Calcium carbonate	CaCO _3_	Soft
Calcium pyrophosphate	Ca _2_ P _2_ O _7_	Medium hardness
Hydroxyapatite	Ca _5_ (PO _4_ ) _3_ (OH)	Medium hardness
Hydrated silica	SiO _2_ · *n* H _2_ O	Medium hardness
Perlite	A mineral silicate	Hard
Alumina	Al _2_ O _3_	Hard

Notably, the amount of toothpaste used for tooth brushing has a significant impact on the cleaning efficacy. 65 Fluoridated toothpastes for children have to be used in small amounts only, due to regulatory and toxicological reasons. 66 It has been shown under standardized *in vitro* conditions that “grain of rice-size” and “pea-size” amounts of toothpaste have a significantly reduced cleaning efficacy compared to lager amounts (i.e., full length of brush; Table 5 ). 65 This study provides evidence that 1 g of toothpaste has efficient plaque removal properties. 65 It should be mentioned that low amounts of fluoridated toothpaste have not been studied regarding their caries preventive efficacy. 51 For fluoride-free toothpastes for children, there is not a limitation in dosage to “grain of rice-size” and “pea-size” amounts of toothpaste. 67 Hydroxyapatite has been shown to be the most versatile nonfluoride active ingredient that works as well or better than fluoride in improving oral health in general. 44 45 67 68

**Table 5:** Cleaning efficacy of different toothpaste amounts *in vitro*

	Dilution degree (toothpaste:water)			
Brushing time (s)	1:1	1:2	1:4	1:8
10	38.5 (±8.4)	32.9 (±4.8)	29.1 (±5.7)	25.7 (±6.0)
30	60.1 (±8.7)	54.5 (±4.4)	35.1 (± 5.7)	33.1 (±6.0)
60	70.1 (±8.6)	66.5 (±3.7)	47.3 (± 5.0)	39.6 (±9.0)
120	77.4 (±5.0)	75.7 (±3.4)	54.1 (± 6.7)	48.2 (±7.1)
180	81.4 (±6.2)	79.2 (±4.9)	62.1 (±5.3)	53.6 (±6.9)
300	85.4 (±4.6)	82.8 (±5.2)	70.6 (±2.3)	60.7 (±5.9)

Source: Data taken from Sarembe et al. 65

Note: Full amount: 1:1 (maximum “full length of brush”); reduced by half: 1:2 (minimum “full length of brush”); one quarter of the amount: 1:4 (“pea-size”); one-eighth of the amount: 1:8 (“grain of rice size”).

Based on literature data, it can be stated that oral care formulations have improved over the years. For example, abrasives have become more efficient, and the use of antibacterial agents is now a common standard in most toothpaste formulations. 50 69

### Antimicrobial Control of the Plaque

Besides the use of abrasives in toothpastes for plaque removal, there is a great variety of surfactants (e.g., sodium lauryl sulfate, sodium methyl cocoyl taurate) and other agents with proven antimicrobial properties used in oral care ( Table 6 ). These agents not only have biocidal effects but also help minimize the growth of plaque (bacteriostatic). Well-known agents are zinc salts, stannous salts, essential oils, and chlorhexidine. Fluoride is also known for its antimicrobial and bactericidal properties. 57

**Table 6:** Overview of antimicrobial agents used in toothpastes to control plaque (ordered alphabetically)

Antimicrobial agents	References
Cetylpyridinium chloride	Marsh 70
Chlorhexidine	Marsh 70
Essential oils (e.g., menthol and thymol)	Marsh 70
Fluoride	Marquis 57
Sodium lauryl sulfate	Marsh 70
Stannous salts	Marsh 70
Zinc salts	Marsh 70

Interestingly, other substances used in toothpastes and mouthwashes such as the commonly used anionic surfactant sodium lauryl sulfate, which is not usually classified as an antimicrobial agent, have an effect on plaque. Shen et al, for example, showed that sodium lauryl sulfate can inhibit the formation of bacterial biofilms. 71

Also, biomimetic actives can be used in oral care. Hydroxyapatite has been shown to reduce the initial plaque formation to enamel surfaces. 72 Additionally, hydroxyapatite acts as acid buffer and reservoir for calcium and phosphate ions in plaque. 6 All these modes of action contribute to hydroxyapatite's clinically proven caries protection. 44 45 67 73 74 Furthermore, natural actives with proven antibacterial/antiplaque action, such as xylitol, 25 polyphenols, 75 and others, 76 can be used as adjunct ingredients. However, side effects of certain antimicrobials have to be considered, such as tooth staining (e.g., in the case of chlorhexidine, stannous salts 77 ), and potential resistances and cross-resistances to antibiotics (e.g., in the case of chlorhexidine 78 ). Additionally, strong antimicrobial agents should not be used for children since they swallow most of the toothpaste, and antimicrobials can have negative impact on a child's health when swallowed. Thus, biomimetic approaches provide a safe alternative. 72 79 Of particular interest is the fact that hydroxyapatite-containing mouthwashes have a significant effect on the reduction and inhibition of plaque formation without killing the bacteria. 72

### pH Buffering of Plaque

Another strategy to reduce the cariogenic potential of plaque is to use active ingredients with pH-buffering effects. Examples are calcium carbonates and (carbonated) calcium phosphates. 27 For example, it has been shown that by using the active ingredient, hydroxyapatite, the pH of plaque can be increased by pH 0.5. 6 Additionally, calcium carbonate and calcium phosphates release calcium ions in the solution, a process that can further help reduce the cariogenic potential of plaque and increase the remineralization of the tooth surface. 27

### Biomimetic Hydroxyapatite and Caries Prevention

Biomimetic hydroxyapatite can be used as active ingredient in caries prevention. 44 45 67 73 74 It has a high biocompatibility, and it is safe if swallowed, thus being an ideal anticaries agent for infants and toddlers. 67 80 Unlike most other potential anticaries agents and fluoride alternatives, hydroxyapatite has been tested in clinical caries trials in comparison with fluorides and was found to be noninferior to fluoride. 44 67 81 The various modes of action of hydroxyapatite listed below contribute to hydroxyapatite's caries preventive efficacy 82 :

- Remineralization, even to deeper layers of enamel and dentin. 83
- Reduction of plaque formation. 72
- Protective layer. 84
- Calcium and phosphate reservoir. 6
- pH-buffering effect. 6
- Cleaning properties. 63

As described in Table 3 , increasing the tooth resistance and remineralization are important factors for caries prevention. While fluorides lead to a superficial surface remineralization, the biomimetic active ingredient hydroxyapatite can also remineralize deeper caries lesions as shown in an *in situ* study using microradiography. 85

## Recommendations for Patients

Caries is still present in our modern society and prevention is important. Based on the studies and review articles presented in this study, the following recommendations can be drawn for patients to prevent caries:

- There are three cornerstones for preventing caries:
  - Remineralization/inhibition of demineralization: increasing tooth resistance.
  - *Plaque removal*
    ○ Using an electric toothbrush and devices for interdental cleaning (e.g., dental floss, interdental brushes).
    ○ Using a sufficient amount of toothpaste (i.e., 1 g).
    ○ The toothpaste should contain (gentle) antimicrobial agents. An alternative to bactericidals is the use of biomimetic agents such as hydroxyapatite for the reduction of plaque formation.
    ○ Additionally, mouthwashes can be used as adjunct to mechanical plaque removal. There is evidence to show the effectiveness of mouthwashes containing essential oils or biomimetic hydroxyapatite in plaque removal.
  - *Diet:*
    ○ Reduction of daily acid and carbohydrate/sugar intake and especially reduction of the frequency of sugar intake.
    ○ Stimulating salivary flow (e.g., by using sugar-free, xylitol-containing chewing gums).
  - *Remineralization/inhibition of demineralization:*
    ○ Using oral care products with remineralizing agents (e.g., fluorides or calcium phosphates).
    ○ Hydroxyapatite is a safe and efficient anticaries agent, which can be used by all age groups including infants and toddlers.
- Regular dental examinations and dental checkups at the dentists are important, especially for high caries risk groups. Frequency of check-ups needs to be made on individual caries risk.

## Conclusion

Caries is caused by the conversion of fermentable carbohydrates by plaque bacteria into acids on the tooth surface. Thus, it is important to focus on sugar reduction and especially on plaque control. Many strategies in caries prevention focus on the remineralization of the tooth surface only, so the two main factors (i.e., sugar and plaque) in the caries process have received less attention in preventive strategies. The reduction of overall sugar intake and the reduction of the frequency of sugar intake is very important to minimize the caries risk. Globally, there are many attempts to reduce the sugar intake such as taxing sugars, dietary advises in schools, etc. In the field of toothpastes, and besides tooth remineralization, plaque removal is an important factor in caries prevention since the presence of plaque is one of the primary factors for caries development. Modern toothpastes contain various types of actives for the removal/control of plaque (e.g., antibacterial agents, abrasives, and pH-buffering agents). One may argue that complete plaque removal cannot be achieved by most patients and, therefore, there is a need for remineralizing agents. Tooth remineralization can be achieved, in part, with fluorides, but perhaps more efficiently with calcium phosphates such as hydroxyapatite and amorphous calcium phosphates. 68 Unlike fluorides, calcium phosphate agents are safe if accidentally swallowed, and calcium and phosphate ions can be released during an acidic attack in the plaque.

In conclusion, modern concepts for caries prevention should focus not only on tooth remineralization and inhibition of demineralization but also on the primary factors of the caries development themselves: sugar reduction (total amount and frequency) and especially efficient removal of plaque from the tooth surfaces during daily oral care.
